# Draft Genome Sequence of *Agrobacterium tumefaciens* Biovar 1 Strain 186, Isolated from Walnut

**DOI:** 10.1128/genomeA.01232-17

**Published:** 2017-11-16

**Authors:** Amisha T. Poret-Peterson, Srijak Bhatnagar, Ali E. McClean, Daniel A. Kluepfel

**Affiliations:** a USDA-ARS Crops Pathology and Genetics Research Unit, University of California, Davis, California, USA; b Microbiology Graduate Group, University of California, Davis, California, USA

## Abstract

*Agrobacterium tumefaciens* biovar 1 strain 186 was isolated from a walnut tree expressing crown gall symptoms. The draft genome sequence of this strain harbored genes for crown gall formation and will be useful for understanding its virulence on Paradox, the predominant hybrid rootstock used for the cultivation of English walnut in California.

## GENOME ANNOUNCEMENT

*Agrobacterium tumefaciens* is a ubiquitous inhabitant of soil and the causative agent of crown gall (CG) disease in plants (1). *A. tumefaciens* enters plants through wounds and then transfers and integrates its plasmid-encoded transfer DNA (T-DNA) into the plant genome (1). In California, English walnut (*Juglans regia*) grown on the hybrid rootstock Paradox (*Juglans hindsii* × *J. regia*) is particularly susceptible to CG, with disease incidence as high as 90% in orchards grown on this rootstock (2, 3). Here, we report a draft genome sequence of *A. tumefaciens* biovar 1 strain 186, isolated from a stem section of a 2-year-old commercial-nursery-grown grafted walnut tree exhibiting CG symptoms (4).

*A. tumefaciens* strain 186 was grown in Trypticase soy broth at 28°C on a rotary shaker (200 rpm) for 24 h. Genomic DNA (gDNA) was extracted from harvested cells using the MasterPure complete DNA purification kit (Epicentre, Madison, WI). Purified gDNA was submitted to MR DNA (Shallowater, TX) for library preparation and 2 × 250-bp paired-end sequencing on an Illumina MiSeq instrument, yielding approximately 8.2 million paired-end reads. After quality trimming and removal of PhiX contamination, raw sequences were assembled with SPAdes version 3.9.0 (5) into 68 contigs, with an *N*_50_ of 731,783 bp and average coverage of approximately 290×. Contigs were aligned to completed genomes of other agrobacteria (RefSeq assembly accession numbers GCF_000092035, GCF_000971595, GCF_000016275, GCF_000442995, and GCF_001551905) using MUMmer version 3.23 (6) to identify the circular and linear chromosomes and plasmid. Bandage version 0.8.1 (7) was then used to manually refine the assemblies and resolve duplications (e.g., rRNA gene operons). The final draft genome consisted of a complete circular chromosome (2.94 Mbp), the tumor-inducing (Ti) plasmid (0.18 Mbp), and linear chromosome (contigs ranging in size from 248 bp to 0.73 Mbp), for a total genome size of 5.72 Mbp and GC content of 59.4%.

The circular chromosome, Ti plasmid, and linear chromosome were annotated via the NCBI Prokaryotic Genome Annotation Pipeline (PGAP) (8), and putative prophage sequences were detected via PHAST (9). PGAP predicted 5,569 genes composed of 5,319 coding sequences, 4 rRNA operons, 54 tRNAs, 4 noncoding RNAs (ncRNAs), and 180 pseudogenes. As expected, the circular chromosome contained homologs of the chromosomally contained virulence genes (two-component system *chvI-chvG* and *chvA*, *chvB*, and *chvE*) (10), and the Ti plasmid harbored a 27.2-kbp region with the *vir* regulon and the oncogenes *iaaM*, *iaaH*, *ipt*, *6b*, and *5* (11). Putative prophages of 33.5 kb and 51.1 kb were detected on the circular chromosome and 0.73-Mbp linear chromosome contig, respectively. This draft genome sequence is being used to provide insight into the unique characteristics of *A. tumefaciens* isolates infecting *Juglans* species.

### Accession number(s).

This draft genome sequence is available in the NCBI database under BioProject PRJNA394767 and GenBank accession number NNAQ00000000. The version described in this paper is the first version, NNAQ01000000.
